# Operationalising Inclusion for Participatory Design: Worked Examples for TRIPOD+AI & PROBAST+AI

**DOI:** 10.1177/11786329261477136

**Published:** 2026-08-19

**Authors:** Riley Martens, Linda Bakunda, Teresa Mushayandebvu, Zack Marshall

**Affiliations:** 1 Department of Community Health Sciences, 70401 University of Calgary Cumming School of Medicine, Calgary, AB, Canada; 2 Department of Precision Health, 70401 University of Calgary Cumming School of Medicine, Calgary, AB, Canada; 3 O’Brien Institute for Public Health, 70401 University of Calgary Cumming School of Medicine, Calgary, AB, Canada; 4 School of Social Work, McGill University, Montreal, QC, Canada

**Keywords:** Artificial intelligence, machine learning, healthcare delivery, health equity, co-design, participatory research, responsible development

## Abstract

Artificial intelligence (AI) and machine learning (ML) are rapidly transforming the healthcare landscape, enhancing diagnostic accuracy, personalising treatments, and improving operational efficiency. However, alongside these advancements comes a critical concern: the potential for AI models to perpetuate, or even exacerbate, health inequities. This paper examines the complex potential of AI in health, and how it can both improve and undermine equity. The key to addressing these challenges lies not only in developing advanced algorithms but in fostering responsible and inclusive development. We argue that to operationalise inclusion in AI systems, research co-design should be prioritised in order to integrate the perspectives of diverse knowledge users, including patients, clinicians, and community partners, shown through worked examples in TRIPOD+AI and PROBAST+AI. This approach encourages future work to rethink the role of knowledge users in the development of AI for healthcare.

## The Role of AI in Healthcare

AI applications are transforming healthcare across clinical and administrative activities including diagnostics, patient monitoring, drug discovery, and resource allocation.^1^ A primary goal of AI in healthcare settings is to support clinical decision-making by leveraging large datasets, aiding medical professionals in diagnosis and treatment planning.^1^ The uptake of this technology in healthcare seems to be driving a paradigm shift, making healthcare systems more proactive rather than reactive.^2^ AI through different use cases has demonstrated the potential to improve the quality of care and health outcomes for patients, partly by decreasing human errors and supporting clinicians to focus on more complex tasks, as demonstrated in diagnostic and surgical domains.^3-5^ This is supported by reduced cognitive burden, automated information retrieval, and procedural assistance with greater accuracy.^4,5^ The results produced by these tools are promising, helping to reduce human bias, expedite clinical trials, and enhance follow-up.^6^ An area that may be overlooked, but is a strong contender for AI innovation, is prognostication. The capacity to predict outcomes such as length of stay, mortality, and transfers to Intensive Care Units (ICU) can reduce the time it takes for patients and clinicians to make decisions. In palliative care, prognostication plays a decisive role in determining treatment pathways and access to hospice settings.^7,8^ In end-of-life care settings focused on patients in the final months of life, mortality prognostication is directly linked to access, with 6 months being the standard for patients in the USA to access hospice care, and to access benefits in the UK^7,9^ Predictions for length of stay and ICU transfer can help with transition planning or goals of care planning, to determine the desired acuity of interventions.^7^ Within the context of prognostic models, this commentary explores the need for research co-design and participatory methods.

## Growing Inequity in AI Related Healthcare

As AI systems are trained and developed, they rely heavily on existing healthcare datasets, which may contain bias.^6^ Historical data often reflects patterns of healthcare disparities, and ML models trained on these data may perpetuate or even amplify these inequities.^10^ These biases can lead to fewer follow-up scans and potentially a greater number of undiagnosed or untreated cancers, worsening health inequity for already disadvantaged populations.^11^ For instance, healthcare data is often skewed toward predominantly white, middle-class populations, and this lack of diversity in datasets can result in AI systems that perform poorly for racialised groups.^11^ The impact of biased AI models is not purely theoretical; real-world examples highlight the dangers of reinforcing inequities. One example is ML models designed to diagnose melanoma that may have different rates of success across a range of skin tones because of underrepresentation in training data.^12^ There is an emerging concern that, without intentional oversight, AI systems could reinforce and exacerbate existing systemic healthcare disparities.^13,14^ Medical AI systems can be shaped by locally majority populations and data infrastructures, which vary across contexts but consistently risk underrepresenting marginalised groups.^15,16^ Widely utilised dataset banks, such as the MIMIC-III clinical database and the eICU Collaborative Research Database are geographically bounded and built from specific healthcare systems which may not be globally generalisable.^17,18^ Most importantly, bias in medical AI is not confined to datasets alone but can emerge throughout the entire development pipeline, including data collection, feature selection, model development, evaluation, deployment, and publication practices.^16^ Biases introduced at different stages may interact and compound, resulting in systematically skewed predictions and clinically unmeaningful outputs for underrepresented groups.^16^ For example, insufficient sample sizes for certain patient populations can lead to systematic underrepresentation of risk and reduced model performance in these groups.^16^

While much of the literature has focused on biases in Western-developed systems, it is important to recognise that medical AI is increasingly being developed across diverse global contexts. In some cases, locally trained models may better reflect regional population characteristics.^14,16^ However, these systems remain shaped by the demographic and structural features of their source data, and therefore may still lack representativeness across other axes, including gender, socioeconomic status, and geography.

As such, the challenge is not solely one of ‘Western’ or white bias, but of broader limitations in the inclusivity and generalisability of health data worldwide. For example, systems that were deployed in South Africa were trained and evaluated using heterogenous multi-country datasets with limited transparency regarding training data composition.^19^ However, when deployed in high burden settings such as rural areas in South Africa, this may introduce mismatch between training distributions and local epidemiological conditions.^20^ Cross-country benchmarking further supports this concern, demonstrating that model performance can vary substantially across different healthcare systems and population groups, which highlights limitations in generalisability beyond the original development contexts.^21^ Centering the discussion of bias in AI models solely in relation to race and ethnicity is insufficient. Existing frameworks such as PROGRESS-Plus emphasise that health inequities arise from multiple intersecting determinants such as race, ethnicity, occupation, gender, religion, education, socioeconomic status, and place of residence.^16,22-24^

## Ethics and Responsible Development in AI

A widely cited and utilised framework for the governance and implementation of emerging technologies is Responsible Research and Innovation (RRI).^25^ One of the four principles underpinning RRI is inclusion.^25^ Inclusion in this context refers to the consideration of interest holders during the ideation and implementation of research and development work.^25,26^ Specifically inclusion factors in those who use and are impacted by the research and innovation process, and is not tied to any particular scientific discipline.^25^ RRI has been mapped to some areas of AI development in healthcare, but to our knowledge never to prognostic models. This presents a gap in literature and a current need for RRI work, as the principles require operationalisation to establish how they work in practical and applied contexts. In this commentary, we operationalise the principle of inclusion to prognostic models as co-design in an attempt to define what it could look like in practice. Co-design is a broad term used in, though not limited to, public health and health services research with occasionally unclear definitions.^27^ Co-design is used intentionally as an umbrella term in this work to capture the broad and varying use of the word and as the principle of inclusion is broad, we propose it aligns with co-design. Keeping co-design broad allows for a higher-level conceptual mapping of inclusion to different participatory methods when concerning AI model development. Co-design denotes a cooperative and inclusionary approach to generating knowledge amongst researchers, clinicians, and others.^27^ In the context of operationalising inclusion, it is referenced as a development and design choice from our perspective. We discuss co-design as a collection of participatory methods, with varying levels of engagement, that pair researchers with interest holders to work together for the purpose of research or innovation. To connect inclusion to co-design and operationalise it in the domain of AI prognostic models, we propose three worked examples to demonstrate how participatory methods can be considered. Research co-design includes associated participatory methods such as community consultation, patient partnership, and research prioritisation. Connecting RRI to AI model development, specifically research co-design and participatory methods, is essential to the intended goals of reducing bias, addressing inequities, and supporting validity. This work aims to begin the process of operationalising RRI principles in prognostic model development, with future work aimed at consolidating the conceptual mapping proposed here.

A way to address biases and inequities is by incorporating ethical analysis at inception and not solely during development or implementation of AI-based applications.^10^ Multiple tools would support this approach including developmental frameworks, building processes, and reporting standards. Utilising these as guiding resources is one strategy to incorporate ethical and reflexive thinking from the beginning of the research and engineering process. Tang and Li’s systematic review highlighted the disconnect between “high-level ethical principles” and empirical research regarding medical AI ethics, underlining the need for transdisciplinary collaboration to ensure that ethical guidelines and standards are put in place for the development, implementation, and utilisation of AI systems.^28^

Applying inclusion through co-design to the development of AI prognostic models, with the involvement of clinicians and other interest holders, can support a more robust development process.^28,29^ By engaging with many interest holders, multiple perspectives are considered, thus ensuring accountability and trustworthiness in innovation.^30^ Co-design can include physicians and nursing staff in healthcare settings, alongside patients, families, and caregivers, integrating both end-users and those directly impacted by their use.^28^ The expertise of clinicians is vital in shaping the design and deployment of tools that are effective and equitable.^28^ However, one interest holder perspective is not representative of all clinicians, allied health providers, or patients.^31^ Ethical considerations in AI-based healthcare applications extend beyond the impact on healthcare providers to include patients and families, groups who are overlooked in some of the current guidelines. Guidelines in the form of reporting standards or evidence of appraisal criteria are strong starting points for incorporating participatory approaches as they can influence the formatting and reporting of evidence. We introduce this topic by first reviewing TRIPOD+AI and PROBAST+AI standards for participatory approaches.^32,33^ As our operationalisation of inclusion is centred around co-design as an umbrella term, corresponding participatory methods are proposed as worked examples to demonstrate their potential in enriching existing standards.

The three worked examples were chosen for their prominence and relevance in participatory literature; however, they are neither exhaustive nor absolute (see Table 1). They were selected to explore what incorporating inclusion from RRI could look like when considering prognostic model development.^25^ TRIPOD+AI and PROBAST+AI were then connected conceptually using directed content analysis with the respective reporting standards as the frameworks being expanded upon.^34^ As this work was intended as a starting point for future scholarship, an exhaustive coding process was not followed. In line with directed content analysis, keywords in TRIPOD+AI and PROBAST+AI were highlighted based on the pre-chosen examples of participatory methods and the author’s expertise.^34^ These sections were then selected for the identified keywords relating to the relevant example. This occurred through frequent re-reading of the TRIPOD+AI and PROBAST+AI standards for the identification of words to be highlighted. This was how the respective sections in TRIPOD+AI and PROBAST+AI were selected for each participatory method. This does not represent an attempt to map all RRI principles to TRIPOD+AI and PROBAST+AI, but as a worked example to direct future research. Following the connection between participatory research activities and the TRIPOD+AI or PROBAST+AI criterion, justification was provided with examples from literature. TRIPOD+AI supports the development of transparent reporting standards for prediction models, while PROBAST+AI is the quality appraisal equivalent to assess.^32,33^ The difficulty of translating conceptual AI ethics into operational guidelines is substantiated by these standards. TRIPOD+AI includes a section for reporting patient and public involvement, which is a development from the previous iteration.^32,35^ Patient and public involvement is an included criterion of the TRIPOD+AI checklist related to the “planning, design, conduct, reporting or dissemination” of the study with involvement of the patient or public should be reported (item 19 on the extended checklist).^32^ Though encompassing multiple, distinct research activities, by limiting the reported inclusion criteria to patient or public involvement, potentially contributes to unreported inclusion of other participatory partners such as community advisory boards, caregivers, families and other interest holders. This lacks contextual specificity where co-design and participatory approaches may not be fully reported. We propose that by incorporating participatory approaches under specific PROBAST+AI and TRIPOD+AI sections, the transparency and validity of the reported work could improve.^32,33^ The following sections will detail recommendations where a specific participatory method can be utilised to enrich the listed items.

**Table 1.** Possible Co-Design Approaches Embedded in TRIPOD+AI & PROBAST+AI^23,33^

Participatory process	TRIPOD+AI section	PROBAST+AI section
Community Consultation	Background – 3.b & c	Participants & Data sources - 1.1 & 1.3
Patient Research Partners	Outcome - 8.a & b	Predictors – 2.1 & 2.2
Research Prioritisation	Usability - 27.a & b	Outcome – 3.1 & 3.4

## Background – Community Consultation

Community consultation can occur in many forms and with many potential groups.^36,37^ We propose co-design with a group based in a specific setting would enrich items 3.b and 3.c (Background) where the intended goals are to describe the target population and describe any health inequalities between sociodemographic groups.^32^ These groups with community expertise can include Community Advisory Boards (CABs), Patient and Family Advisory Councils (PFACs), and Research Advisory Groups (RAGs).^30,37^ A community advisory board is a group intended to advocate and represent a specific community.^38^ Consulting with a specialised group with expertise informed by technical and lived experience would strengthen goals of describing the intended population and any known existing health inequalities.^38^ The community board would be capable of helping establish a stronger understanding and description of a community or population. The board would be able to highlight elements not readily apparent in literature, particularly in understudied areas. Additionally, understanding and documenting health inequalities can require a strong knowledge of socio-economic determinants of health (SEDOHs), which may not always be available to researchers.^14,38^ Consultation with a community advisory board could address this gap by exchanging expertise and experience, making the prognostic model more contextually relevant. Patients were recruited to form a RAG, where they co-created survey questions and contributed to research questions with the study team in building a mortality prediction model for the end goal of building trust in AI applications.^30^ Community consultations represent a participatory approach capable of enhancing the background in model development as determined by TRIPOD+AI.^32^ Community consultation is a robust approach for including interest holders, however there are specific conditions and limits to it. Previous examples of CABs demonstrate the importance and value boards add to research based on feedback by inspiring iterative changes to all facets of the process.^39^ Challenges do exist with CABs and incorporating community consultation, including communication, organisation, and role clarity.^39,40^ A noted challenge beyond logistical, is institutional.^41^ Funding may not be attributed directly to patient partners or community co-investigators.^41^ This can present limitations both logistically and institutionally.

Regarding PROBAST+AI, community consultation would be most relevant to 1.1 & 1.3 (Participants & data sources).^33^ Community consultation could provide knowledge related to SEDOHs, issues with pre-existing data sources, and caveats with participant eligibility criteria. A CAB would draw on previous experience related to sampling and data collection.^42^ Community consultation could strengthen data provenance with the identification of challenges and barriers. This is a strong consideration given the potential for research fatigue in communities.^43^ Community consultation could direct efforts of the research team in order to not contribute to research fatigue or harm the relationship between community and academia.^43^ Community consultation is particularly salient when considering eligibility criteria. If a prognostic model is designed for community or outpatient settings, SEDOHs could have a large influence and should be considered for training.^14^ Community consultation is necessary for the validity and relevance of the SEDOH data, but also direction as there may be stigma or value judgements around the data.^42^

## Outcome – Patient Partners

Sections 8.a and 8.b (Outcome) provide a strong rationale for the inclusion of patient partners on the study team.^32^ These sections are relevant to describing the outcome across sociodemographic factors and supporting subjective interpretation of data. Related to these elements are SEDOHs where lived experience may provide a strong capacity to establish in-depth insights and rationales for outcomes and group differences.^42^ The added perspective of a patient partner can serve to strengthen the epistemic work of the research team, particularly in areas that are subjective or ambiguous.^30^ A patient partner from a RAG was able to identify adherence to prescribed medication as a factor to the accuracy of a prediction model, which the researchers had not considered previously.^30^ Patient partners, inclusive of families or caregivers, would strengthen the work outlined in the Outcome reporting standard of TRIPOD+AI.^32^

Items 2.1 and 2.2 (Predictors) on PROBAST+AI are strong contenders for including patient partners on the study team.^33^ Lived experience in clinical settings and care pathways could be invaluable for robustness and comprehensiveness in predictors. PROBAST+AI outlines potential issues if predictors are not defined, assessed, and pre-processed for all participants in the same manner.^33^ PROBAST+AI supports acknowledging that these steps may occur differently for certain participants due to setting or systemic marginalisation.^33^ Patient partners on the study team would have the expertise to recognise this difference and direct the study team accordingly. Particularly for any SEDOHs, contextualising data, variables, and assessments in community is critical as there may be value-based judgements in these decisions.^42^ Having patient partners able to identify sources of bias, stigma, or contextual differences can better direct the use and analysis of said data.^14,42^

## Usability – Research Prioritisation

Sections 27.a and 27.b (Usability), describing information pertinent to applicability and usefulness of the model is required to be reported in the TRIPOD+AI checklist.^32^ As every novel model regarding implementation is a proposed change to the pre-existing workflow and care context, research prioritisation is appropriate for collecting perspectives and considering concerns.^44,45^ Though potentially not as involved as a community advisory board for consultation or engaging patient partners on the study team, priority setting is a robust starting point for understanding the current workflow, tools used, and concerns of end-users.^44,46^ This method is a participatory approach more in the immediate or short-term time horizon, typically completed through interviews, surveys or focus groups as opposed to long-term relational consultation like a CAB.^47^ As clinicians and healthcare professionals are typically the intended end-users for prognostic models, their input should shape the research.^44^ This research prioritisation could reduce wasted efforts or redundant work to determine if a model is needed or helpful in a clinical setting.^44^ More specifically, a needs assessment, an approach for collecting input and insight into a proposed change, is a salient method for incorporating co-design into model development.^46,48^ Unless a model is being developed solely for technical innovation with no intention of being clinically deployed, a needs assessment is an important aspect of implementation mapping.^49^ A potential tool to consider is the Shinners Artificial Intelligence Perception tool, a validated assessment to gauge the perception of AI among healthcare professionals.^50^ By collecting perspectives from interest holders, the usability of models can be contextualised in the corresponding setting.

Regarding PROBAST+AI, research prioritisation could be embedded into 3.1 & 3.4 (Outcome) to support the validity of definitions, assessments, and the time intervals between predictors and outcomes.^33^ PROBAST+AI states that subject matter expertise may be required for the items as outcome definitions, assessments and time intervals can be clinically complex.^33^ Research prioritisation from health professionals could be a robust avenue to collect the needed expertise.^44^ In terms of model development, subject matter expertise would be critical in determining requirements, usability, and future deployment. Without adequate prioritisation, models could risk contextual irrelevance and unfeasibility in clinical workflows. Subject matter expertise could support the validity of outcomes chosen as outlined by PROBAST+AI.^33^ Research prioritisation would also direct what problems there are to be solved and feasibility concerns for use and implementation, potentially resulting in higher quality evidence and reporting. An example for predictive models is the time interval from predictor to outcome may not be the correct length for the model to properly classify the results.^33^ This is an example of how assessing the priorities of interest holders could contribute to better validity. There may be limitations to how applicable literature and other evidence is to local contexts and specific care pathways. Thus, research prioritisation with interest holders could address that gap.

## Participatory Research as Responsible Development

Embedding participatory processes in prognostic model development would strongly benefit from co-design. Participatory research and associated methods encompass many strategies, but co-design is an established approach to model development that includes the active involvement of interest holders in the construction and implementation of technology.^27^ Specific co-design processes proposed for prognostic model development include meaningful engagement with patient partners, research prioritisation, and consultation with a community advisory board.

AI development must be informed by diverse perspectives to ensure that models are fair and representative.^11^ Diverse teams are more likely to ask critically relevant questions and create systems that are inclusive and fair.^13,28^ For example, when Black female scientists audited AI-based facial recognition systems, they identified significant biases, highlighting the importance of diversity in tech development.^51^ By involving patients and healthcare providers, especially from marginalised groups, in the design, testing, and implementation of AI tools, the technologies can be better tailored to serve populations and their concerns.^51^ Diversity among both patients, researchers, and clinicians in this work is vital.^52^ However, a caveat for participatory methods is that engagement should not be limited to patients or clinicians, rather this extends to family, caregivers, and any member of the public who will be directly impacted by model development.^52^ This approach fosters trust and ensures that tools can be effectively adapted to meet the needs of specific communities.^11^ Over time, this can contribute to narrowing the healthcare equity gap, improving outcomes for underrepresented groups, and fostering a more inclusive healthcare ecosystem.^14^

## Calls for Participatory Design Processes

Several studies call for more participatory design in AI development.^26,28,53^ Some argue that involving end users, particularly from diverse backgrounds, is crucial to identifying issues that might otherwise go undetected, helping to prevent real-world harm.^54^ Certain aspects to highlight from these calls to action include the necessary interprofessional collaboration of healthcare, relevance of SEDOHs, and contextualising in communities.^52,55^ Lam et al. emphasise the importance of empowering end users to lead their own audits of AI systems, ensuring that potential algorithmic harms are detected from the perspective of those directly impacted.^56^ Additionally, Ovalle et al. highlight the Queer in AI community, which advocates for the adoption of intersectional, community-led, participatory design to foster inclusivity and challenge exploitative practices in AI development.^57^ These studies collectively highlight the need to adopt participatory design processes in order to create more ethical, equitable, and effective AI systems.^54,56,57^ Despite the inherent advantages of participatory research demonstrated in healthcare settings, adaptation to AI-specific contexts can be challenging.^27,55^ This is due to the technical nature of AI, the opacity of models, and the training of model designers.^10,14,26^ These nuances need specific attention in the co-design of AI-based applications for healthcare.

## Limitations of Participatory Research

Though the benefits of co-design are strong and represent a shift in model development, participatory approaches also present multiple concerns. Challenges including exclusion, tokenism, and power imbalances are well-documented issues with participatory approaches. Exclusion, closely linked with tokenism, occurs when interest holders are not represented in the participatory process. One group of interest holders may be incorrectly assumed to be representative of all invovled.^58,59^ An example regarding model development would be clinician consultation, where only physicians are considered, whereas nurses and allied health are excluded. Tokenism relates to performative or symbolic acts done for patient engagement, which undermines the intent of participatory methods.^58^ Power imbalances can also be present between researchers and participatory partners. This can undermine the intention and efficacy of participatory approaches.^60^

There are some stipulations due to AI and health as a domain, where these concerns can reflect epistemic limitations of participatory research. Power imbalances exist for data analysts compared to other interest holders or researchers because they may have more influence due to technological literacy and understanding of the model systems.^55^ These inequities can be exacerbated by the opacity of models and ethical disengagement of developers.^55^ Some designers may be aware of the considerable implications and potency of ethics in healthcare, but others assign most of the responsible decision making and patient safety to physicians.^55,61^ This power imbalance inherent in participatory processes can be exacerbated by technological literacy, opacity, and designer awareness and can escalate to become more than challenges to the point of becoming epistemic limitations.

## Considerations Regarding Participatory Research

Factors that influence the capacity for interest holders to provide insight into topics can impact on the feasibility of participatory approaches, particularly co-design. Some drawbacks of participatory research have already been highlighted; these factors present potential limitations and were selected due to their significance to feasibility. Technological literacy, transparency, and designer pedagogy can all be epistemic limitations when conducting participatory methods in prognostic model development. Other considerations for participatory approaches that also deserve to be mentioned include investment in infrastructure and tying research funding to participatory outcomes. These are two strong elements for promoting a greater uptake in participatory methods; however, rather than highlighting conceptual challenges, they focus on logistical or structural challenges.

### 1.1. Digital Literacy

A challenge to participatory research strategies in AI-based work is digital literacy.^26^ Previous knowledge of AI may give some individuals participatory advantages in research as digital literacy can act as a barrier to interest holder engagement.^26^ A series of focus groups demonstrated that patients engaged in consultation for AI-research typically are unfamiliar with more technical concepts.^26^ This knowledge gap needs to be accounted for in the design and application of AI-based tools, even when using strategies designed to reduce inequities. Resources and strategies can be developed to help address this challenge but centering them around the concept of technological literacy can tie interventions together.^26,52^ There are additional methods and assessments to gauge technological and AI literacy in addition to offering a suite of resources.

There have been proposed strategies to promote digital and AI literacy. Some of these methods include using software to explain how the models work, teaching less complex models first such as generalised linear models, and developing educational resources.^26,30^ Another potential solution to this issue is to provide a standardised training module about AI to all participants.^26^ The module included information regarding ethical challenges, technical concepts, and roles AI can play in medicine.^26^ This was found to increase understanding of the content matter and enhance the participation of patients in co-design activities.^26^ This type of supplemental education may be required to enhance the effectiveness of participatory research in AI. Otherwise, participatory research may be less effective in addressing inequities and representing all interest holders as technological literacy has been identified as a factor multiple times.^26,30,52,62^

### 1.2. Transparency

Beyond the required digital literacy to understand AI and its capabilities, the opacity of machine learning and associated models also presents a feasibility challenge to participatory research. Opacity can refer to technological literacy, but also to the unknown functioning of models and how outputs are produced.^63^ This opacity undermines trust and hinders the adoption of AI technologies in healthcare, as both practitioners and developers remain unaware of the processes behind diagnostic outcomes.^61,64^ In response, transparency has been proposed to reveal and clarify how these models work. Transparency is a broad term meant to capture accountability, safety, and informed consent.^65^ For the proposed taxonomy, explainability and interpretability are methods to assess the goals of transparency.^65^ A key component of transparency is explaining how these tools work for different audiences. In healthcare, this applies to the end-users (clinicians) and those impacted (the patients). There are proposed layers of accountability to direct the transparency of models.^65^ The insider layer relates to AI developers, the internal is for healthcare professionals, and external transparency is marked for patients.^65^ The technical explanation and conceptual understanding of a model is important for engineers and designers, but for the end-users and those impacted, explaining how a model works is imperative for informed consent.^64,65^ There are varying levels of risk regarding AI integration in patient care.^66,67^ A lower risk example would be AI helping to automate administrative workflow compared to higher risk diagnostic models analysing radiographic images.^67^ Levels of opacity regarding machine learning outputs from patient data can contribute to a risk of unfair treatment and worsening health outcomes.^68^ The argument for transparency towards patients extends from informed medical consent.^65^ To consent to a procedure or treatment, patients must have understanding thereof.^65^

An absence of transparency, encompassing both explainability and interpretability, could be an epistemic limitation to co-design as opacity can impact multiple interest holders across different layers.^65^ Participatory methods centering patients would benefit from transparency measures, but so would any that involve clinicians, allied health or other interest holders. This fosters understanding in interest holders including patients, physicians, and other healthcare professionals.^30^ Simultaneously, applying participatory research to AI development in healthcare makes the process and knowledge generation more transparent. One area for addressing transparency for AI in healthcare is Explainable AI or XAI, which is a way to provide clarity and understanding of how machine models function.^68^ This helps establish trust between patients and clinicians.^64^ However, there are tradeoffs between the explainability and the accuracy of the models due to the complexity.^62^

### 1.3. Preparing Designers in Parallel

Just as participatory research requires researchers to prepare to partner with patients, co-design encourages designers to work in tandem with a range of interest holders. Preparing designers for a participatory approach is essential for ensuring successful partnerships.^53^ Designers have different perspectives compared to researchers or patients, and do not always consider moral concepts or teaching from areas beyond computer science.^69,70^ There are multiple frameworks for teaching AI ethics to future developers.^69,71^ Some include incorporating philosophy or other inter-disciplinary education, while others highlight specific ethical principles.^69,71^ Participatory research often highlights the need to engage with interest holders and end-users, but it is also important to prepare designers for the participatory approach.

Having designers of systems and models unprepared for co-design can be a barrier to participatory research. Without training and support, designers’ participatory approaches risk being improperly conducted, with concerns of missing interest holders or tokenism.^58^ If a research team is not interdisciplinary and composed of only designers with no participatory training, the capacity to recruit or engage different interest holders will be a challenge.^45^ An acknowledged strength of a research team was the cross-training between clinical care and data science regarding the study team members and associated clinicians.^30^ It was noted that patient engagement is not particularly incentivised for research team members, and this could be extended to the training of designers and developers.^30^ A component of preparing designers in parallel is the interdisciplinary and transdisciplinary challenge. As designers can work in many knowledge domains and with different groups, having an interdisciplinary understanding of co-design is warranted to embed these practices into research. Co-design and other participatory approaches have been used in many different areas.^27,30^ Training regarding its effectiveness could promote greater uptake across disciplines beyond AI prognostic model development.

A series of interviews conducted with AI health developers and designers in industry concluded awareness and recognition of ethical challenges was a needed skill.^55^ This arises from the need for greater education and awareness to prepare designers and developers for ethical assessment and navigation skills. This is further supported by a recent review of AI ethics pedagogy in post-secondary contexts.^72^ The review’s findings included that the majority of AI ethics education is embedded within technical courses with little empirical or competence-based evaluation.^72^ This directly demonstrates there is a tangible need for competence-based AI ethics pedagogy for designers and developers.^72^ Participatory processes could be a pragmatic and relevant component to include.

## Conclusion

The integration of AI and machine learning in healthcare holds significant potential for improving diagnostics, treatment, and overall patient outcomes. However, as these technologies evolve, careful attention is required to mitigate the risks. AI systems, when trained on biased data, can inadvertently perpetuate existing healthcare disparities, particularly among marginalised populations. This highlights the importance of inclusive co-design and participatory research to ensure that AI tools are developed with diverse perspectives in mind, especially those most impacted by health inequities. Engagement with the public and community has been largely overlooked in the development of AI-based applications in healthcare. Addressing challenges such as digital literacy, transparency, and developer pedagogy concerns will be critical to building trust and ensuring equitable outcomes. Given the conceptual nature of this manuscript, the proposed arguments and action points have not been validated by a use case, and only through worked examples and conceptual justification. This commentary marks the beginning of the operationalisation process, future work will progress this conceptual mapping and validate it through more data-driven processes. As this example of directed content analysis was done only by an individual, reproducibility would be enhanced by a second reviewer in future scholarship. The identification of potential starting points for participatory methods by incorporating them into existing guidelines is a strength of this work. Future recommended areas for research are operationalising the other RRI principles for prognostic development or further validation work surveying interest holders. Additional implications for future reporting include incorporating the worked examples proposed for TRIPOD+AI and PROBAST+AI, including more explicit detail around inclusion, and revised reporting standards. There is a parallel need to prepare designers to work with patients, community groups and members of the public on co-design initiatives. By fostering collaboration between developers, clinicians, and patients, AI technologies can better serve all populations and help narrow healthcare equity gaps.
